# Genetic aetiology of global developmental delay and intellectual disability in Africa: a scoping review

**DOI:** 10.3389/fgene.2026.1718279

**Published:** 2026-03-26

**Authors:** Norbert Dukuze, Janvier Hitayezu, Jeanne P. Uyisenga, Olivier Hakizimana, Vincent Bours, Annette Uwineza, Abdullateef Isiaka Alagbonsi

**Affiliations:** 1 Center for Human Genetics, School of Medicine and Pharmacy, College of Medicine and Health Sciences, University of Rwanda, Kigali, Rwanda; 2 Department of Biochemistry, Molecular Biology and Genetics, School of Medicine and Pharmacy, College of Medicine and Health Sciences, University of Rwanda, Huye, Rwanda; 3 Center for Human Genetics, Centre Hospitalier Universitaire Sart-Tilman, University of Liege, Liege, Belgium; 4 Department of Paediatrics, University Teaching Hospital of Kigali (CHUK), Kigali, Rwanda; 5 Department of Biology, College of Science and Technology, University of Rwanda, Kigali, Rwanda; 6 Department of Physiology, School of Medicine and Pharmacy, College of Medicine and Health Sciences, University of Rwanda, Huye, Rwanda

**Keywords:** Africa, chromosomal abnormalities, epigenetics, global developmental delay, intellectual disability

## Abstract

**Background:**

The genetic aetiology of global developmental delay (GDD) and intellectual disability (ID) in Africa is poorly understood. This review synthesises the available information on this topic.

**Methods:**

Original articles published in the English language between January 2000 and June 2024 on the African population were included. Literature was retrieved from PubMed, Scopus, and Web of Science, in accordance with the PRISMA guidelines.

**Results:**

Of all the 54 African countries, only 13 reported the genetic factors associated with GDD and ID. The genes related to GDD and ID were reported in Egypt (22), Tunisia (17), Morocco (16), South Africa (10), Algeria (4), Sudan (3), Libya (2), Nigeria (2), Rwanda (4), Mali (1), Cameroon (1), DRC (2), and Tanzania (1), although some genes were reported in more than one African country. At least 45 genes associated with GDD and ID have been reported in the African population, whereas 21 genes associated with these disorders are yet to be documented in Africa.

**Conclusion:**

This review provides, to the best of our knowledge, the first comprehensive review of the genetic aetiology of GDD and ID in Africa. It presents an imbalance of gene research on GDD and ID across African regions, with North African countries dominating this field of study.

## 1 Introduction

### 1.1 Background

In normal development, the central nervous system develops during the early stages of embryonic life and continues for years until adulthood (Gaitanis and Tarui, 2018). During early infancy, a rapid process acceleration occurs that continues into maturity, involving various processes such as dendritic pruning, myelination, and the development of a vast and intricate network of connections (Spear, 2013). Global developmental delay (GDD) and intellectual disability (ID) are vast categories of syndromes in which delays in certain developmental milestones affect various areas of development, like motor skills, language, cognitive abilities, and social skills (Bélanger and Caron, 2018). The GDD is defined as the inability to attain the desired developmental milestones within the anticipated age range, especially in children under the age of 5 years (Vasudevan and Suri, 2017). The worldwide prevalence of ID is estimated to be 2%–3%. It is characterised by significant impairment in both intellectual functioning and adaptive behaviour before the age of 18 years (Jacquemont et al., 2014). The American Psychiatric Association’s DSM-5 defines ID as a defect in intellectual functioning and adaptive behaviour that begins in the developmental period and affects three areas of daily life, including the conceptual domain, which encompasses knowledge, reasoning, memory, and the ability to write, read, and perform mathematics; the social domain, which describes social interactions including friendships, communication, empathy; and the practical domain, which includes personal care, organising daily life, going to school, having a job, and managing finances (Regier et al., 2013).

Both GDD and ID are prominent features of neurodevelopmental disorders (NDDs). However, their formal diagnosis is made when the intellectual quotient is below 70% (AlMutiri et al., 2023). The severity of GDD and ID is classified as mild, moderate, severe, and profound. They vary greatly and can manifest alone or in conjunction with congenital abnormalities or other neurological disorders, including epilepsy, sensory impairment, and autism spectrum disorders (ASD) (Burnside et al., 2011). The ID can be caused by multiple factors, including severe malnutrition, infections, complicated deliveries, and maternal alcohol abuse during pregnancy, but the majority of cases are also known to be caused by genetic factors (Huang et al., 2016). Globally, in paediatric primary care, GDD and ID are among the most frequent causes of patient referrals (Aldosari and Aldosari, 2024). The ID can be isolated or syndromic and present with clinical symptoms like hypotonia, delayed speech, and seizures (Cousin et al., 2021). Over the past decades, it is believed that autosomal dominant ID predominates in outbred nations like the United States of America and Western Europe, but autosomal recessive ID has some preponderance in the Middle East and some parts of northern Africa, where consanguinity is a common practice (Fridman et al., 2021). Discoveries and advancements in new technologies like next-generation sequencing have brought the ability to analyse individual genomes and find a vast array of genetic variants (Satam et al., 2023). In the East African region, a high prevalence of neurodegenerative diseases has been recently reported (Onohuean et al., 2022).

While it is established that ID is a type of NDD, recent studies have shown that it increases the risk of developing neurodegenerative diseases due to the interaction of genetic, environmental, biological, and social factors, and neurodegenerative diseases can worsen the impact of ID on the patient’s quality of life (Lamptey et al., 2022; Shateri and Tahan, 2025). For instance, ID patients with Down syndrome may develop symptoms of Alzheimer’s disease later in life owing to the presence of the amyloid precursor protein (APP) gene on chromosome 21 (Fortea et al., 2020; Martínez-Cué and Rueda, 2020). Also, ID leads to enhanced oxidative stress and neural inflammation, which result in nerve damage (Halliwell, 2001; Teleanu et al., 2022). This comorbidity causes diagnostic and management overlaps that hinder precision and personalized care by healthcare specialists.

Different methods, including whole-exome sequencing (WES) and copy number variants (CNVs) testing, are used to diagnose patients with GDD and ID. About half of patients with GDD and ID have a mutation that causes the disease, and single-nucleotide variations (SNVs) and CNVs are both considered to be important contributors to these disorders (Li et al., 2024). Over the past few decades, there have been reports linking more than 600 genes containing over 130 unusual SNVs and CNVs to both GDD and ID (Banerjee et al., 2022). The mode of inheritance for GDD and ID differs from X-linked, autosomal recessive, or autosomal dominant, and *de novo* mutations (Pande et al., 2023). Combining genetic testing for GDD and ID has been suggested to reduce the diagnostic odyssey, elaborate the etiologic diagnosis, lay the groundwork for identifying novel early diagnostic biomarkers, and help to design successful intervention targets (Zhang et al., 2024).

### 1.2 Significance and objective of the study

Recently, our team published a review that documented 61 genes responsible for the aetiology of ASD in the African population. The data therein showed that 26 genes were identified using a polymerase chain reaction (PCR)-based method, 22 genes were identified using sequencing technologies, and 12 genes and one *de novo* chromosomal aberration were identified through other techniques. However, no African study has identified any ASD gene with genome-wide association studies (GWAS), while at least 20 ASD risk genes reported in non-African countries have yet to be confirmed in Africa (Hakizimana et al., 2024). Beyond genetics, our team also presented a comprehensive review of the environmental factors associated with microcephaly, another form of NDD (Izabayo et al., 2026), highlighting the fact that NDDs arise from environmental factors, in addition to genetic factors. Though some progress has been made in developing countries, there remains an obvious knowledge gap about the genetic aetiology of both GDD and ID in Africa. People in Africa are greatly impacted by these conditions, but a thorough knowledge of the genetic aetiology is still lacking (Baine-Savanhu et al., 2023). Globally, there is a lack of ethnic diversity in the genetic aetiology of GDD and ID, due to the scarcity of data from the African continent, which is known to have a lot of ethnic diversity (Gomez et al., 2014). Thus, by identifying and synthesising data from previous studies on the genetic cause of GDD and ID in the African population, this scoping review hopes to fill some knowledge gaps and guide future investigations in this important field.

However, achieving the aim of this review may not be possible without some intersection with neurodegenerative diseases, as they share some genetic factors in common. Thus, some genetic factors known to be associated with neurodegenerative diseases are mentioned in this review to the extent of their involvement in GDD and ID. Furthermore, our review encompasses both GDD and ID, as the former is diagnosed in children under 5 years and may progress to the latter, which is diagnosed after 5 years. While some overlaps exist in the genetic aetiology of syndromic (presence of other clinical features in addition to ID) and non-syndromic ID (presence of ID only without other clinical features), there are reports demonstrating genes specific to either of them. It is noteworthy that it is clinically challenging to rule out some subtle neurological or psychiatric disorders in patients with non-syndromic ID, as the cognitive impairment may hinder their diagnosis. Thus, the distinction between syndromic and non-syndromic ID is often blurred. To enable a comprehensive documentation of genetic factors in the African population, our review incorporates both syndromic and non-syndromic forms of ID, without much distinction.

## 2 Methods

### 2.1 Search strategy

The scoping review was conducted using the Preferred Reporting Items for Systematic Reviews and Meta-Analyses (PRISMA) standards (Page et al., 2021; Adepoju et al., 2025; Ajayi et al., 2025; Kampire et al., 2025; Onohuean et al., 2025). The search technique involved querying large databases, including PubMed, Scopus, and Web of Science. The combinations of the following keywords were included in the search terms: “dysmorphology”, “Africa”, “genetic mutations”, “global developmental delay”, “intellectual disability”, chromosomal abnormalities”, and “epigenetics”. The search was expanded to include the names of all 54 African countries to find more studies that satisfied the eligibility criteria. The search strategy used sets of general search phrases with “AND” in each database to find genetic variations associated with GDD and ID in Africa. The initial search terms were “global developmental delay OR developmental delay OR mental retardation OR intellectual disability” together with a collection of search phrases including “genetic study OR next-generation sequencing OR genomic study. Studies published between January 2000 and June 2024 were included in the review. The review focused mainly on articles published in English. Since we expected a sizable amount of the study to have been conducted in cooperation with other researchers and institutions, we also included studies conducted outside of Africa. Articles that discussed only the environmental causes of GDD and ID were not included.

### 2.2 Selection process and quality assessment

The relevance of the identified studies’ titles and abstracts was checked independently by two authors. The full-text articles were then examined against the inclusion criteria. Where necessary, a third author was consulted to settle disagreements between the two authors, and consensus guided the final selection of the studies. This scoping review followed 5 steps to conduct a systematic review (Khan et al., 2003). The inclusion criteria include that the article must be original research and published in a peer-reviewed journal, report a phenotype or a genetic cause of GDD or ID, and the study participants must have come from an African population (Munn et al., 2014). Two authors independently evaluated each study by using the checklist, and each study was given one of three possible overall quality scores: “good,” “average,” or “poor”. Articles that were judged average or poor using the appraisal methods were eliminated, and disagreements were settled through consensus debate.

### 2.3 Data extraction

Data were extracted using a standardised and piloted form (Hakizimana and Alagbonsi, 2025b; Hakizimana and Alagbonsi, 2025a; Hakizimana et al., 2025; Izabayo et al., 2026; Ndinganire et al., 2026), including study features like author, country, year of publication, sample size, patient demographics, molecular genetic techniques, and genetic variants linked to GDD and/or ID.

## 3 Results

### 3.1 Selected articles

**FIGURE 1:**
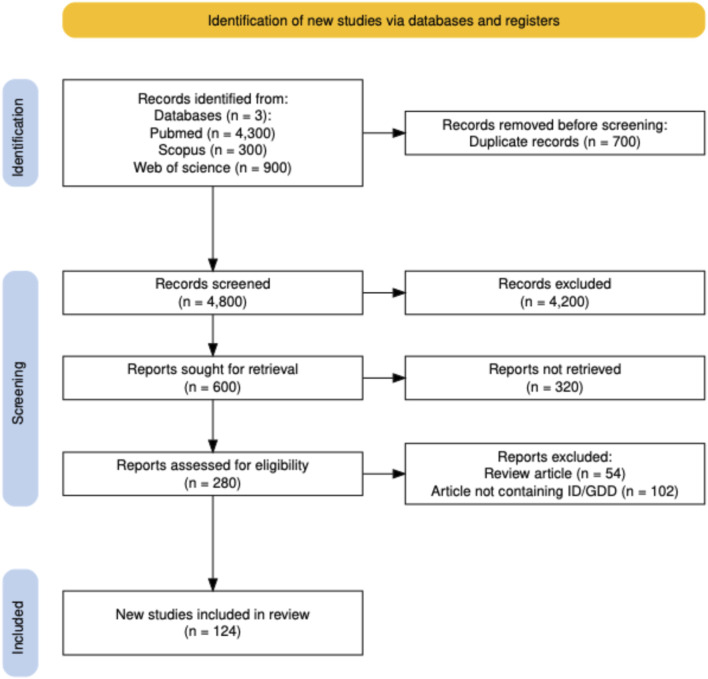
PRISMA flow chart of the article screening and selection process.

The three databases searched in this study identified 5,500 papers, out of which 700 records were removed for being duplicates, resulting in 4,800 articles. By screening the titles of the 4,800 publications, 4,200 records were also excluded based on irrelevance to the research questions. Then, the abstracts of 600 papers were reviewed, out of which only 124 full-text articles were obtained and read after removing papers not describing original research, letters to the editor, and articles not reporting the phenotypes of GDD and/or ID. Of these 124 eligible articles, 87 (70.16%) were case reports, 27 (21.77%) were case series reports, 5 (4.03%) were cohort studies, and the remaining 5 (4.03%) were prevalence studies (Figure 1).

### 3.2 Patient’s characteristics

Conforming to the fact that GDD and ID are generally diagnosed in childhood, the majority of the publications focused on the paediatric population (93.55%, 116/124), out of which 20 also reported on both adult and paediatric patients. Only a small portion of articles focused on the adult population (4.84%; 6/124) and prenatal diagnosis (1.61%; 2/124). There were many different conditions in the dataset, which were divided into three groups: single gene disorders, chromosomal abnormalities (aneuploidy, large structural change, CNVs), and imprinting disorders. Most of the reports focused on single-gene disorders (69%; 31/45 genes), and the remaining 31% were chromosomal abnormalities as reflected in the testing strategies used.

### 3.3 Countries where genetic factors for GDD and ID have been reported

**FIGURE 2:**
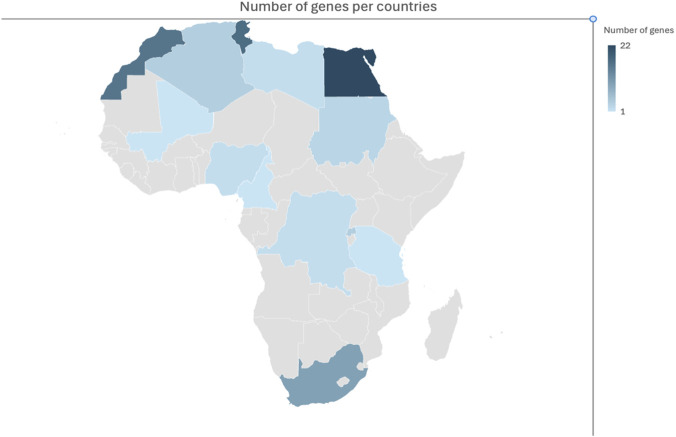
Distribution of reported genes across Africa.

Of all the 54 African countries, only 13 reported the genetic factors associated with GDD and ID. Northern African countries predominated the publications, where Egypt had the largest contributions (17.74%; 22/124), followed by Tunisia (13.71%, 17/124). Other countries in the Northern African region are Morocco (16), Algeria (4), and Libya (2). In sub-Saharan Africa (SSA), South Africa has the highest publications (8%; 10/124). Other countries are Nigeria (2), Sudan (3), Rwanda (4), Mali (1), Cameroon (1), DRC (2), and Tanzania (1). Egypt, Tunisia, and Morocco are the top 3 countries, representing 44% of the genes included in this review. In summary the number of genes identified in this review were from the following countries: Egypt (22), Tunisia (17), Morocco (16), South Africa (10), Algeria (4), Sudan (3), Libya (2), Nigeria (2), Rwanda (4), Mali (1), Cameroon (1), DRC (2), Tanzania (1). These figures show that some genes were reported from more than one African country (Figure 2).

### 3.4 Techniques used to identify the genes associated with GDD and ID in Africa

#### 3.4.1 Sequencing technologies

##### 3.4.1.1 Genes identified with WES

The WES was the most utilised method for identifying pathogenic or likely pathogenic variants across different African nations (Table 1). In Egypt, WES revealed pathogenic mutations in *UBE3A, ZEB2, HEPACAM, ASPM, PGAP3*, and *SLC39A8* (Glotov et al., 2023). Similar studies in Tunisia revealed variants in *UBE3A, MeCP2, RAF1*, *CCDC82, CHD7, PAK2*, and *VPS13B* (Fendri-Kriaa et al., 2012). In Morocco, WES identified causative variants in *OCRL, ASPM, DYM, ZEB2*, *METTL23*, and *MLL2*; the same method was used to identify genes causing Mowat-Wilson Syndrome in patients with ID (Fu et al., 2022). Novel mutations in *the MLC1* and *HEPACAM* genes were identified in 12 Egyptian patients with Megalencephalic leukoencephalopathy (Abdel-Salam et al., 2016). From Tanzania, compound heterozygous variants in *EVC2* were detected (Dekker et al., 2019). In Rwanda, WES revealed mutations in VPS51 in siblings with GDD and microcephaly (Uwineza et al., 2019). In South Africa, WES and linkage analysis identified *DXS424* (Xq24) and DXS548 (Xq27.3) in patients with X-linked severe mental retardation (Schroer et al., 2010). A study done in Tunisia using WES identified a novel UBE3A frameshift mutation in the exon 16 coding region in patients with ID and epilepsy (Abaied et al., 2010). The clinical spectrum of patients with Sjögren-Larsson syndrome and a novel ALDH3A2 mutation from Egypt was also reported by using WES (Abdel-Hamid et al., 2019). K243Ifs*X15* causes Bardet-Biedl syndrome, a single founder mutation, and was found in patients from South Africa by using Sanger sequencing and WES. This is a multisystem disorder characterised by obesity, polydactyly, ID, and loss of vision due to a progressive retinopathy (Fieggen et al., 2016). Different genes, including *ESCO2* causing Roberts syndrome in Egyptian patients, *RAF1* gene causing Noonan syndrome in Tunisian patients, and *FGFR3* (Ser252Trp), all of these patients presented with ID. Different genes associated with ID and GDD, like *VPS13*B in Cohen, *EVC2* in Ellis-van Creveld, MeCP2 in Rett, and *TBCE* in Sanjad-Sakati syndrome, were found in the African population, and the founding mutations were also identified mostly by using WES. The same technique was used to identify a novel WDR62 variant, c.390G > A, in 2 Sudanese patients with microcephaly and GDD (Kim et al., 2023). The FA2H gene mutation was reported among patients with hereditary spacistic paraplegia 35 in Mali using WES, a type of next-generation sequencing (Landouré et al., 2019).

**TABLE 1:** Genetic markers and associated symptoms.

No	Gene	Country	Method used	Genetic diseases	No of people tested	Authors
1	*UBE3A*	Tunisia	WES, PCR	Angelman syndrome	14	Abaied et al. (2010)
2	*MYO5A*	Egypt	Sanger sequencing	Griscelli syndrome	*3*	Abd Elmaksoud et al. (2020)
3	*ZEB2*	Egypt	WES	Mowat-Wilson syndrome	1	Abdalla and Zayed (2014)
4	CAG expansion in the form of a smear sized 69–75 repeats	Egypt	PCR	Spinocerebellar ataxia type 2 (SCA2)	1	Abdel-Aleem and Zaki (2008)
5	*ALDH3A2*	Egypt, Algeria	PCR, WES	Sjögren–Larsson syndrome	35	Abdel-Hamid et al. (2019)
6	*MLC1* and *HEPACAM*	Egypt, Tunisia, Morocco, Libya	WES	Megalencephalic leukoencephalopathy	8	Abdel-Salam et al. (2016)
7	*ASPM*	Egypt, Morocco, Algeria	WES	Molecular and phenotypic spectrum of ASPM-related primary microcephaly	37	Abdel-Hamid et al. (2016)
8	*PGAP3*	Egypt, South Africa	Sanger sequencing, WES	Hyperphosphatasia with mental retardation syndrome	10	Khalifa et al. (2025)
9	*FGFR3*	Egypt, Tunisia, South Africa	Sanger sequencing, WES, PCR	Muenke syndrome with pigmentary disorder and probable hemimegalencephaly	1	Abdel-Salam et al. (2011)
10	*PQBP1*	Egypt, Tunisia	Sanger sequencing, PCR	PQBP1-related intellectual disability	4	Abdel-Salam et al. (2018)
11	9p24p12	Morocco	FISH, CGH-Array	Insulin-like growth factor type 1 deficiency with *de novo* inverted duplication 9p24p12 and developmental delay	1	Amasdl et al. (2016)
12	*WDR62*	Sudan, Morocco	WES	Primary microcephaly	2	Naseer et al. (2017), Dekker et al. (2019)
13	*SLC39A8*	Egypt	WES	Autosomal-Recessive Intellectual Disability with Cerebellar Atrophy Syndrome	6	Boycott et al. (2015)
14	*EDNRB*	Morocco, South Africa	Sanger sequencing	Hirschsprung disease, microcephaly, and mental retardation (Goldberg-Shprintzen syndrome)	4	Mowat et al. (1998)
15	t(6; 10)(q27; q25.2)	South Africa	CGH-Array	Severe mental retardation	57	Brusnicky et al. (1986)
16	*OCRL*	Morocco	WES, Sanger sequencing	Oculo-cerebro-renal Lowe syndrome	1	Hadjiu et al. (2020)
17	Homozygous *AHI1* gene mutation (p.Thr304AsnfsX6)	Morocco	CGH-Array	Joubert syndrome	3	Chafai-Elalaoui et al. (2015)
18	*DXS424* (Xq24) and DXS548 (Xq27.3)	South Africa	Linkage analysis, WES	X linked severe mental retardation	26	Christianson et al. (1999)
19	*SLC2A1*	Algeria	Sanger sequencing	De Vivo disease. GLUT-1 deficiency syndrome	1	Daoudi et al. (2014)
20	*EVC2* gene, c.653_654del, p.Val218Glyfs*12 in exon 5, and c.2710C>T, p.Gln904* in exon 16	Tanzania	WES	Ellis-van Creveld syndrome	1	Dekker et al. (2019)
21	c.1878delA of the *DYM* gene	Morocco, Egypt	WES, Sanger sequencing	Dyggve-Melchior-Clausen syndrome	1	Chafai-Elalaoui et al. (2015)
22	K243Ifs*X15 BBS*	South Africa	Sanger sequencing, WES	Bardet Biedl syndrome	76	Fieggen et al. (2016)
23	Novel mutation c.695 G > TMeCP2	Tunisia, Morocco, Egypt	WES	Rett syndrome	1	Mietto et al. (2025)
24	Microduplications, 15q and Xq	Tunisia, Egypt, South Africa	CGH-array, MLPA	Moderate mental retardation	8	Hila et al. (2010)
25	*ESCO2*	Egypt	WES, PCR	Robert syndrome	16	Malla et al. (2016)
26	*RAF1 *c.776C > A (p.Ser259Tyr)	Tunisia	WES	Noonan syndrome	1	Malla et al. (2016)
27	*FGFR2* (Ser252Trp)	Nigeria	Sanger sequencing	Apert syndrome	1	Fanganiello et al. (2007)
28	*FMR 1*	Cameroun, South Africa, Egypt, DRC, Tunisia	Sanger sequencing	Fragile X syndrome	46	Mbachu et al. (2024)
29	*TBCE* gene on chromosome 1q42-q43	Tunisia, Libya, Morocco, Sudan, Egypt, Algeria	WES	Sanjad-Sakati syndrome	1	Kerkeni et al. (2015)
30	*FA2H* gene causing *SPG35*	Mali, Morocco	WES	Hereditary spastic paraplegia type 35	1	Landouré et al. (2019)
31	4p16.3 deletion of 3.47 Mb	DRC, Egypt	CGH-Array, FISH	Wolf-Hirschhorn syndrome	1	Mbuyi-Musanzayi et al. (2017)
32	Novel (p.Y54X) Nonsense Mutation in the iduronate-2-sulfatase (intellectual disability) gene	Rwanda	WES	Hunter Syndrome	2	Mutesa et al. (2007)
33	*De novo* 19p13.2p13.12 deletion	Morocco	CGH-Array	Overgrowth syndrome and severe developmental delay	1	Natiq et al. (2014)
34	13q interstitial deletion	Morocco, Nigeria	CGH-Array	Hereditary retinoblastoma and intellectual disability	1	Outtaleb et al. (2020)
35	*CHD7*	Morocco	WES	CHARGE syndrome	1	El Yajouri and Mahraoui (2018)
36	*MLL2*	Morocco, South Africa, Egypt, Tunisia	WES	Kabuki syndrome	1	Makrythanasis et al. (2013)
37	*PAK2*	Tunisia	Sanger sequencing, PCR, WES	Mental retardation with neuropsychiatric features	1	Werren et al. (2024)
38	*VPS13*B	Tunisia	WES	Cohen syndrome	1	Rejeb et al. (2017)
39	*METTL23*	Morocco	WES	familial mild intellectual disability with dysmorphic features	1	Smaili et al. (2020)
40	Partial trisomy 10q and monosomy 4q	Egypt, Tunisia	CGH-Array	blepharophimosis mental retardation	1	Bartholdi et al. (2008)
41	*CCDC82*	Sudan, Egypt	WES	Syndromic intellectual disability	6	Yahia et al. (2024)
42	Chromosomal abnormalities	Tunisia	Standard cytogenetics	non-syndromic mental retardation	1,420	Bouhjara et al. (2012)
43	CNVs ranged from 0.9 Mb–34 Mb	Rwanda	CGH-Array	development delay/intellectual disability with multiple congenital anomalies	50	Uwineza et al. (2014)
44	*VPS51*	Rwanda	WES	Microcephaly with brain malformation	2	Uwineza et al. (2019)
45	47, XX,+del(9)(q11), 46,XY,del(13)(q34) and 46,der(22)t(10XX; 22)	Rwanda	Karyotype	Global Developmental Delay, Intellectual Disability and/or Multiple Congenital Anomalies	664	Uwineza et al. (2016)

##### 3.4.1.2 Genes identified through Sanger sequencing

Sanger sequencing was used in several single-gene investigations and confirmed mutations in *MYO5A* ***,*** *FGFR3* ***,*** *PQBP1* ***,*** *EDNRB* ***,*** *FMR1, GLUT-1* ***,*** *FGFR2* ***,*** *DYM, BBS*, and *PAK2* across Egypt, Morocco, Algeria, Mali, South Africa, Nigeria, and Cameroon (Table 1). The results from these studies suggested a higher prevalence of GDD and ID in African patients and demonstrated the value of admixture analysis and African genetic diversity in understanding the aetiology of NDDs. The same technique was used in Egypt to diagnose children with GDD and microcephaly, and the identified gene was *ASPM* (Oegema et al., 2020). Egyptian children with GDD were found to be associated with abnormal, elevated serum levels of phosphorus and recessive mutations in genes involved in the glycosyl phosphatidylinositol pathway, including *PGAP3,* which was identified by Sanger sequencing (Khalifa et al., 2025). Muenke syndrome with pigmentary disorder and probable hemimegalencephaly was reported for the first time in Egypt by using Sanger sequencing (Abdel-Salam et al., 2011). Two studies using Sanger sequencing in Morocco identified the EDNRB and OCRL genes as causing ID (AitRaise et al., 2022). A patient from Nigeria with Apert syndrome was found to have a mutation in fibroblast growth factor receptor 2 (Ser252Trp) by using Sanger sequencing, and GDD was among the clinical manifestations (Kana et al., 2018). A Moroccan study found Joubert syndrome, a rare congenital disorder characterised by brain malformation, GDD with hypotonia, ocular motor apraxia, and breathing abnormalities, and the mutations found in these patients were a homozygous mutation (p.Thr304AsnfsX6) in the AHI1 gene (Chafai-Elalaoui et al., 2015). An Algerian patient with developmental delay and myoclonic seizure was diagnosed with GLUT 1 deficiency by using Sanger sequencing, and SLC2A1 was the responsible gene (Klepper et al., 2020). The primary transporter involved in the cellular absorption of glucose into numerous tissues is glucose transporter type 1 (Glut1), which is primarily expressed in erythrocytes and the brain.

#### 3.4.2 PCR-based methods

PCR-based techniques were less frequently used, though 7 genes were identified using these methods, which proved useful for detecting known variants or repeat expansions (Table 1). In Egypt, PCR identified *ALDH3A2* mutations (Liu et al., 2020). Furthermore, CAG repeat expansions (69–75 repeats) were validated by methylation-sensitive PCR as the cause of both GDD and ID (Malik et al., 2021). Egyptian studies confirmed mutations in *FGFR3* and *PQBP1,* and they are known to cause hypochondroplasia and X-linked ID, respectively (Prinos et al., 1995). In Tunisia, the *PAK2* gene mutation was found to be associated with ID using PCR and Sanger sequencing-based methods (Abdelwahed et al., 2020). A patient from Mali was found to have a mutation in the *FA2H* gene, known to cause hereditary spastic paraplegia SPG35 (Landouré et al., 2019). A *de novo* 9p24 to p12 was found in a Moroccan patient with GDD. By using CGH-array and PCR, several genes were identified, especially insulin-like growth factor binding protein (Amasdl et al., 2016). The Fluorescence *In Situ* Hybridisation (FISH) and PCR were used in Egypt to identify deletions in the *ESCO2* gene (EL Hawary et al., 2022). The UBE3A was also identified using PCR, in addition to WES (Abaied et al., 2010).

#### 3.4.3 Other molecular-based techniques

Five different molecular techniques, including Comparative Genomic Hybridisation Microarray (CGH-Array), Multiplex Ligation-dependent Probe Amplification (MLPA), FISH, Karyotyping, and Linkage analysis, identified different genes causing GDD and ID in Africa (Table 1). CGH-Array played a significant role in uncovering chromosomal microdeletions and duplications, and pathogenic CNVs were detected in 9p24p12, 19p13.2p13.12, and 13q deletions (Karim et al., 2023). CGH-array also identified deletions of 4p16.3 in patients with Wolf-Hirschhorn Syndrome from the Central African Republic (South et al., 2008). Array comparative genomic hybridisation was performed in Rwandan patients with ID and multiple congenital abnormalities; most of the sizes of CNVs ranged from 0.9 Mb to 34 Mb (Uwineza et al., 2014). Partial trisomy 10q and monosomy 4q were associated with ID by using the CGH array. In Morocco, a patient with *de novo* inverted duplication 9p24p12 was reported, and the patient presented with GDD and insulin-like growth factor type 1 deficiency (Amasdl et al., 2016). MLPA confirmed microduplications of 15q and Xq in Tunisian patients with GDD and ID (Miclea et al., 2021). Linkage analysis in South Africa was used to map loci on Xq24 and Xq27.3 in patients affected by X-linked mental retardation (Choo et al., 1984). CAG expansion in the form of a smear-sized 69–75 repeats was found in Egypt by using Methylation studies; these patients were diagnosed to have Spinocerebellar ataxia type 2 (SCA2**)** associated with ID (Abdel-Aleem and Zaki, 2008). Novel (p.Y54X) nonsense mutation in the iduronate-2-sulfatase (IDS) gene was reported in Rwandan patients with Hunter syndrome (Mutesa et al., 2007). 47, XX,+del(9)(q11), 46,XY,del(13)(q34) and 46,der(22)t(10XX; 22) were reported in patients with ID and congenital malformations, A newborn with partial monosomy 10q25.2; and congenital malformations was reported from South Africa and the monosomy derived from a balanced maternal translocation t(6; 10)(q27; q25.2) (Sabnis et al., 2021).

### 3.5 Genes reported in non-African populations that are yet to be identified in Africa

A total of 21 unique genes associated with GDD and ID were identified to have been reported exclusively in studies conducted outside of Africa, but have not been reported in Africa (Table 2). The most common method of gene identification was WES, used in 17 out of the 21 genes discovered. Sanger sequencing was used in a smaller subset, notably for the validation of ACTB and ACTG1 variants, both reported in China and Poland, respectively. The non-African countries and the genes identified were United States (*ADSL*, *ZMYND11*, *SMC3*, *SON),* Germany (*AP1S2* and *ATP1A3)* France (*AMER1),* China **(** *ACTB* and *TELO2*), Poland (*ACTG1*), Iran (*ASNS)* Italy (*ARG1),* Iceland (*ATRX),* Australia (*BCAP31),* Canada (*UNC80),* England (*USP9X),* Belgium (*WWOX),* Japan (*TBCK),* Switzerland (*NONO),* and Sweden (*BRAT1).* Several of these genes (*ATRX*, *AP1S2*, *USP9X*, *NONO*) are located on the X chromosome, suggesting a notable contribution of X-linked inheritance to NDDs in those non-African populations (Brand et al., 2021).

**TABLE 2:** List of genes associated with GDD and ID reported in non-African populations but not yet identified in Africa.

No	Genes	Countries	Method used	Author
1	*ACTB*	China	Sanger sequencing	Nie et al. (2022)
2	*AMER1*	France	WES	Boutet et al. (2010)
3	*ACTG1*	Poland	Sanger sequencing	Dawidziuk et al. (2022)
4	*ADSL*	USA	WES	Mouchegh et al. (2007)
5	*AP1S2*	Germany	WES	Saillour et al. (2007)
6	*ARG1*	Italy	WES	Russo et al. (2024)
8	*ASNS*	Iran	WES	Russo et al. (2024)
9	*ATP1A3*	Germany	WES	Rosewich et al. (2012)
10	*BRAT1*	Sweden	WES	Henry et al. (2025)
11	*ATRX*	Iceland	WES	Amorim et al. (2016)
12	*BCAP31*	Australia	WES	Whalen et al. (2021)
13	*ZMYND11*	USA	WES	Yates et al. (2020)
14	*UNC80*	Canada	WES	Obeid et al. (2018)
15	*USP9X*	England	WES	Homan et al. (2014)
16	*WWOX*	Belgium	WES	Piard et al. (2019)
17	*TELO2*	China	WES	Zhao et al. (2023)
18	*TBCK*	Japan	WES	Miyamoto et al. (2021)
19	*SMC3*	USA	WES	Ansari et al. (2023)
20	*SON*	USA	WES	Kandaswamy et al. (2020)
21	*NONO*	Switzerland	WES	Mircsof et al. (2015)

## 4 Discussion

This scoping review provides an overview of the genetic causes of GDD and ID in African populations, focusing on a range of genes and disease mechanisms implicated in these conditions.

### 4.1 Molecular and pathophysiological mechanisms used by the identified genes to cause GDD and ID

Human brain development follows conserved spatiotemporal patterns across mammals. It starts with neurulation, a process in which the neural plate, specified from embryonic ectoderm, folds and fuses to form a closed neural tube. This tube then segments into lineage-restricted vesicles that become the forebrain, midbrain, and hindbrain (Zhou et al., 2024). Neuroepithelial stem cells line the interior wall of the neural tube and populate the proliferative ventricular zone, then transform into radial glial cells that produce postmitotic excitatory and inhibitory neurons and subsequently glia (Miranda-Negrón and García-Arrarás, 2022). Mammalian neurogenesis relies on intermediate progenitor cells, the immediate descendants of radial glial cells that divide in the subventricular zone to amplify neuronal production (Pontious et al., 2007). In the developing cortex, precursors of excitatory neurons migrate radially along the radial glial scaffold of radial glial cells to populate the cortical plate in a stereotypical ‘inside-out’ manner, with late-born upper-layer neurons migrating past early-born deep-layer neurons (He et al., 2015). Inhibitory interneurons arise mostly from progenitors in the ganglionic eminence and migrate tangentially into the cortex (Hernández-Miranda et al., 2010). Upon establishment of neuronal networks, dendritic spines form to enable neuronal communications via synapses. Synaptic assembly and later dendritic and synaptic pruning are highly plastic and neuronal activity-dependent, and their continuous refinement persixp22 into young adulthood (Gipson and Olive, 2017).

The GDD and ID can result from the disruption of neuronal circuit formation and connectivity involving important genes controlling axon guidance, synapse function, and cytoskeleton organization (Liaci et al., 2021). They are primarily caused by genetic abnormalities in synapse formation and plasticity (Washbourne, 2015). In recent years, many of the most common single-gene mutations for both GDD and ID were shown to be sufficient to cause cellular deficits and to recapitulate relevant synaptopathies in patient-derived organoids. Examples include *CACNA1C* in Timothy syndrome (Miclea et al., 2015). The *CACNA1C* gene encodes the α1C subunit of the L-type voltage-gated calcium channel, which is pivotal in mediating calcium influx in response to membrane depolarization. Calcium signalling is essential for various neuronal processes, including gene transcription, neuronal excitability, and synaptic plasticity (Kessi et al., 2021). The *FMR1* gene encodes the fragile X messenger ribonucleoprotein 1, an RNA-binding protein that regulates the translation of specific mRNAs at synapses and plays a critical role in synaptic development and plasticity by modulating the local synthesis of proteins necessary for synapse formation and function (Malecki et al., 2020). In the absence of FMRP, there is dysregulated protein synthesis at synapses, leading to abnormal dendritic spine morphology and impaired synaptic plasticity. These alterations disrupt the formation and refinement of neural circuits, contributing to the cognitive and behavioural deficits associated with the fragile X syndrome (Sidorov et al., 2013).

*MECP2* encodes the methyl-CpG-binding protein 2, which functions as a transcriptional regulator by binding to methylated DNA and modulating gene expression. It is highly expressed in mature neurons and is critical for maintaining neuronal function and synaptic stability (Zhao et al., 2013). Neuronal activity induces phosphorylation of MeCP2 at serine 421, a modification that influences its ability to regulate genes involved in dendritic growth and synaptic maturation, such as brain-derived neurotrophic factor. Mutations in *MECP2* disrupt this regulatory mechanism and lead to impaired dendritic development and synaptic function (Bellini et al., 2014). UBE3A encodes an E3 ubiquitin ligase for voltage-dependent big potassium channels in cytoskeletal processes such as adherens junctions and centrosome genes (Delgado-Ramírez and Rodríguez-Menchaca, 2019). It plays a vital role in synaptic development and plasticity by regulating the turnover of synaptic proteins (Alvarez-Castelao and Schuman, 2015). Ubiquitination is a key posttranslational modification for the controlled protein degradation and proteostasis. The substrate specificity is determined by a family of E3 ubiquitin ligases, which are encoded by more than 600 genes in the mammalian genome. Gain- or loss-of-function of many E3 genes results in neurodegeneration or NDDs, affecting synapse function (Kawabe and Stegmüller, 2021).

Chromatin remodelling and the proper assignment of epigenetic marks on the genome are of fundamental importance for brain ontogenesis. These processes are also key control points in the stepwise transition from pluripotency to neural precursors to terminally differentiated neurons and glia, and are involved in developmental events such as neuronal migration and connectivity formation (Jakovcevski and Akbarian, 2012). Deficiency of fatty acid dehydrogenase (FALDH) activity results from ALDH3A2 loss-of-function mutations. It is believed that the neurotoxic effect of aldehyde buildup affects myelination and neurodevelopment, which leads to ID (Rizzo, 2014). *The SLC39A8* gene, located on chromosome 4q24, encodes the manganese transporter ZIP8, and its detrimental variants cause a type 2 congenital disorder of glycosylation and ID (Bonaventura et al., 2021). Iduronate 2-sulfatase (IDS) is involved in the lysosomal degradation of the glycosaminoglycans heparan sulfate and dermatan sulfate. It is localized at Xq28 distal to the fragile X site, and it causes ID, especially in microdeletions of Xq (Wilson et al., 1990). MYO5A, a gene encoding the myosin motor protein, has been linked to a variety of NDDs, including GDD and ID in different populations. Myosin VA is involved in melanosome transport as well as exocytosis of neuropeptides and other substances from brain neurons that support synaptic activity (Hódi et al., 2006). A study that used Sanger sequencing in an Egyptian patient with Griscelli syndrome showed that MYO5A affects neuronal transport and intracellular signalling, which in turn disrupts brain development and leads to GDD or ID (Abd Elmaksoud et al., 2020). Similarly, research conducted on European populations, Canada, and Asia has identified certain mutations in MYO5A linked to GDD and ID, frequently with more phenotypic descriptions (Aldosari and Aldosari, 2024).

The *ZEB2* gene encodes a transcription factor involved in neuronal development. It is associated with Mowat-Wilson syndrome, which is characterized by features of GDD, ID, and different forms of congenital malformations (Birkhoff et al., 2021). A neo mutation c.776T>C of the OCRL gene causes oculo-cerebro-renal Lowe syndrome as a rare X-linked disorder caused by the inositol bisphosphate 5-phosphatase deficiency (Zhang et al., 2021). By interacting with various components of the splicing machinery and other regulatory proteins, PQBP1 plays a critical role in neural progenitor proliferation, neuronal differentiation, and synaptic integrity. Its dysfunction can lead to alterations in gene expression profiles that manifest as ID, microcephaly, and other NDDs (Waragai et al., 1999). 9p24p12 inverted duplication and insulin-like growth factor 1 (IGF1) insufficiency are linked to the inverted duplication of 9p24p12, which causes GDDand dysmorphic traits. The IGF1 is essential for the growth and development of the brain. Its absence causes synaptogenesis and neuronal proliferation to be disrupted, which leads to GDD (Amasdl et al., 2016). The t(6; 10)(q27; q25.2) translocation, a balanced translocation involving 6q27 and 10q25.2, was linked to severe ID. These breakpoints likely disrupt gene regulatory elements critical for brain development, such as the FOXO3 and DMBT1 genes 017). A 3.47 Mb deletion at 4p16.3 causes Wolf-Hirschhorn Syndrome (WHS), a condition characterized by GDD, facial dysmorphism, and seizures. Important genes include WHSC1 and LETM1, which are involved in mitochondrial function and chromatin modification, respectively. The WHS phenotype is caused by altered neuronal excitability and synaptic function (Mbuyi-Musanzayi et al., 2017).

A *de novo* deletion of 19p13.2p13.12 has been linked to overgrowth syndrome and severe GDD. Brain growth and cell proliferation are altered when regulatory genes are lost. Overgrowth, macrocephaly, and cognitive impairment are the results of disrupted growth signals (Redin et al., 2017). Partial Trisomy 10q and Monosomy 4q cause ID and blepharophimosis. Important genes located on this section are overexpressed or lost as a result of dosage imbalance. Disrupted craniofacial and neuronal development causes facial anomalies and GDD (Popescu et al., 2021). Microdeletions have been linked to non-syndromic ID in Tunisia, and these deletions affect single critical neurodevelopmental genes. Haploinsufficiency of genes like *SYNGAP1* involved in synaptic plasticity leads to impaired learning and memory due to disrupted signalling in glutamatergic neurons (Mircsof et al., 2015). CNVs (0.9–34 Mb) with congenital anomalies were linked to ID, as the CNVs disrupt gene networks involved in organogenesis and brain wiring. Widespread deletions/duplications lead to multiple congenital defects and GDD (Shaikh, 2017). Complex karyotypes (47,XX,+del(9)(q11), 46,XY,del(13)(q34), 46,del(22)t(10; 22), and multiple chromosomal rearrangements involving chromosomes 9, 13, and 22 are linked with GDD and congenital anomalies and the structural changes lead to gene loss, position effects and chromosomal instability (Tomac et al., 2020). The 15q11-q13 region is associated with Angelman/Prader-Willi spectrum, and when disrupted, each of these disorders results from the loss of function or over-expression of at least one imprinted gene. As impaired synaptic maturation and plasticity lead to ID (Kalsner and Chamberlain, 2015), CAG repeat expansion (69–75 repeats) in ATXN2 causes Spinocerebellar Ataxia type 2 (SCA2), Moreover, toxic gain-of-function due to expanded polyglutamine tracts in the ATXN2 gene induces neuronal degeneration in the cerebellum, brainstem, and cortex, which leads to GDD (Costa et al., 2024).

Neural stem and progenitor cells drive neurogenesis, which is controlled by numerous molecular characteristics unique to humans via a variety of routes, including transcription modulation, cell cycle regulation, signalling pathways, mitochondrial dynamics, and metabolism (Garone et al., 2024). The involvement of *PGAP3* and *EDNRB* in neurodevelopmental pathways further highlights the possibility of population-specific interactions and mutations that lead to GDD (Da’as et al., 2020). Depending on the ethnic background and consanguinity within groups, some *EDNRB* polymorphisms that produce distinct phenotypes may contribute to a larger or lower burden of developmental problems in African populations (Zhong et al., 2005; Campbell and Tishkoff, 2008). The *ASPM* gene is a major cause of primary microcephaly, as abnormal spindle-like microcephaly (ASPM) is essential for normal mitotic spindle function in embryonic neuroblasts (Zhong et al., 2005), and *ASPM* mutations lead to abnormal cell division in the brain, resulting in reduced brain size and significant cognitive impairments, which are commonly associated with ID (Garrett et al., 2020). *FGFR3* is a family of polypeptide growth factors involved in a variety of activities, including mitogenesis, angiogenesis, and wound healing, and is well-documented to cause ID in different populations (Keegan et al., 1991). *MLC1* and *HEPACAM* mutations disrupt the functioning of glial cells, mostly astrocytes, leading to impaired brain myelination and cognitive deficits, which serve as the basis for GDD (López-Hernández et al., 2011). The *CHD7* gene is associated with CHARGE syndrome, and *MLL2,* related to Kabuki syndrome, is linked to both GDD and ID. They both affect the neurogenesis mechanism together with the *PAK2* gene, which causes ID (Martin, 2015). The *WDR62* gene is located on chromosome 19q13.12, and its mutation disrupts mitotic spindle function in neural progenitors, leading to premature differentiation and a reduced number of neurons, which causes microcephaly and ID (Bhat et al., 2011). Homozygous *AHI1* gene mutation (p.Thr304AsnfsX6) is also associated with GDD and ID. For instance, *AHI1* encodes jouberin, which localizes to the basal body of cilia and is essential for cilia-mediated signalling pathways crucial for proper brain morphogenesis. The loss of function mutations leads to impaired cellular signalling, which induces cerebellar and midbrain malformations (Chafai-Elalaoui et al., 2015).

The DXS424 and DXS548 are highly polymorphic markers located on the X chromosome; they are not genes, but they are located near the FMR1 gene (Xq27.3), which is mutated in Fragile X syndrome, the most common inherited cause of ID (Basuta et al., 2015). Mutations in the SLC2A1 gene hinder passive glucose transport across the blood–brain barrier, and they are the primary cause of GLUT1-deficient syndrome (Vulturar et al., 2022). The EVC2 gene (Ellis-van Creveld syndrome 2) is located on chromosome 4p16, and its mutation indirectly affects neurodevelopment through abnormal hedgehog signalling, which affects brain patterning (Galdzicka et al., 2002). *DYM* gene causes Dyggve-Melchior-Clausen disease (DMC), a rare autosomal recessive disorder associated with spondyloepimetaphyseal and ID (Elalaoui et al., 2011). Bardet-Biedl Syndrome (BBS) is a genetically heterogeneous disorder, and the mutation of its gene induces disruption of the BBSome complex, which is crucial for intraflagellar transport and causes ID (Priya et al., 2016). ESCO2 on chromosome 8p21.1 encodes an acetyltransferase essential for establishing sister chromatid cohesion during the S phase of the cell cycle. Loss-of-function mutations disrupt cohesin complex acetylation, which induces genomic instability and ID (Whelan et al., 2012). RAF1, located on 3p25.2, is activated by RAS-GTP binding at the cell membrane. Gain-of-function mutations in *RAF1* enhance MAPK signalling, which leads to uncontrolled cellular proliferation and ID (Tran et al., 2021). *FGFR2* is located on 10q26, where it encodes a receptor tyrosine kinase involved in cell proliferation, migration, and tissue repair. Its mutation induces premature fusion of cranial sutures and defective neurodevelopmental signalling (Azoury et al., 2017).

The *FMR1* gene is located at Xq27.3 and encodes for fragile X messenger ribonucleoprotein 1, an RNA-binding protein responsible for mRNA transport and localization at synapses. Its mutation leads to the disruption of normal brain development and function, which is common in fragile X patients (Malecki et al., 2020). The *TBCE* gene encodes a protein responsible for folding and assembly of α- and β-tubulin; mutations are associated with a syndromic form of ID, like Sanjad–Sakati syndrome (Ghawil et al., 2024). *FA2H* is located at 16q22.3 and encodes an enzyme that adds a hydroxyl group at the 2-position of fatty acids in sphingolipids, the main components of myelin, and the mutation leads to disruption of the myelin sheath and neurodegeneration (Alderson et al., 2004). The deletion of the long arm of chromosome 13 results in 13q deletion syndrome, which has different presentations, including ID, delayed speech, and hypotonia. *VPS13B*, located at 8q22.2, is involved in vesicle-mediated protein sorting and intracellular trafficking in the Golgi apparatus and endosome (Duplomb et al., 2014). Methyltransferase-like protein 23 (*METTL23*) is located at 17p11.2, and loss-of-function mutations affect chromatin remodelling and abnormal neuronal development (Zha et al., 2024). The *CCDC82* gene, located at 11q21, encodes a protein with coiled-coil domains responsible for protein–protein interactions, and the mutations lead to abnormal protein interactions and ID (Riazuddin et al., 2017). Large CNVs are a major cause of syndromic and non-syndromic ID, and when a functional copy of a gene is deleted, it leads to insufficient gene dosage (Shearer et al., 2014). The *VPS51* gene, located at 11q13.2, is a component of the Golgi-associated retrograde protein complex, and its mutations impair lysosomal enzyme trafficking and neuronal synaptic dysfunction, which lead to ID (Gershlick et al., 2019).

### 4.2 Conclusion

This scoping review identified at least 45 genes that have been reported to be associated with GDD and ID in the African population, while 21 genes associated with these disorders are yet to be documented in Africa. This study provides, to the best of our knowledge, the first comprehensive review of the genetic aetiology of GDD and IDin Africa. It also identified diseases associated with these genes and the mechanisms (molecular and pathophysiological) by which these genes lead to GDD and ID. Furthermore, it presents an imbalance of genetic research on GDD and ID across African regions, with North African countries dominating this field of study. It is, therefore, imperative to improve genetic testing capacity to diagnose both GDD and ID in Africa. It is also important to conduct studies on the genetic aetiology of GDD and ID in the remaining 41 African countries, to enable the identification of other genes likely associated with these disorders in a genetically- and ethnically-diverse population like Africa. This study is limited by its inability to differentiate genetic factors based on syndromic and non-syndromic forms of ID, owing to the overlap between them. It is also limited based on the inability to isolate genetic factors associated with ID that are independent of neurodegenerative diseases, also due to their comorbidity or the development of the former into the latter. Finally, this study does not account for environmental factors associated with GDD and ID, even though such factors have been documented for microcephaly in a previous study. We, therefore, recommend future studies that will carefully address these limitations, building on the strengths of our current study. Finally, the use of GWAS and whole-genome sequencing in African genomic studies is highly recommended for enhanced identification of the genomic variants and regulatory proteins associated with NDDs.

## Data Availability

The original contributions presented in the study are included in the article/supplementary material, further inquiries can be directed to the corresponding authors.
